# A silent threat: Post-traumatic rib fragments near the ascending aorta

**DOI:** 10.1007/s12471-025-02013-7

**Published:** 2026-01-14

**Authors:** Ana Rodrigo Costa, Catarina Lencastre, Glória Cabral Campello

**Affiliations:** 1 Serviço de Cardiologia, Unidade Local de Saúde do Tâmega e Sousa, Penafiel, Portugal; 2 Serviço de Medicina Intensiva, Unidade Local de Saúde do Tâmega e Sousa, Penafiel, Portugal

**Fig. 1:**
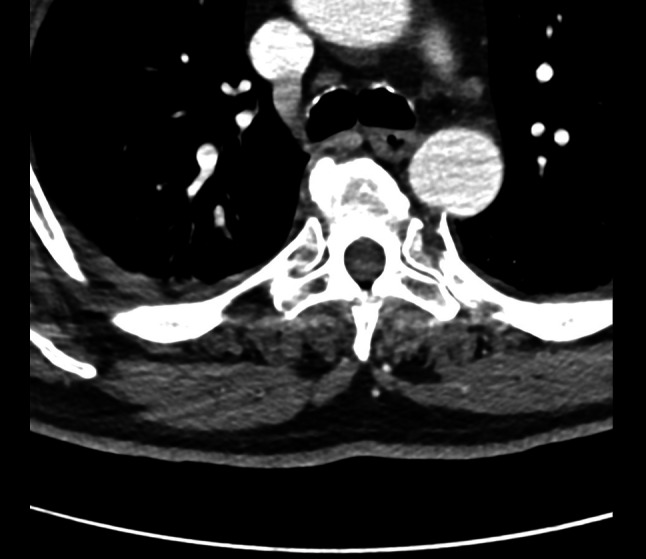
5th left rib in contact with the posterior wall of the descending aorta

A 55-year-old man was admitted to the intensive care unit following a high-energy polytrauma sustained in a bicycle-versus-car road traffic accident. Upon arrival at the emergency room, he was hemodynamically stable, with a GCS score of 15 and no signs of active bleeding. A bedside echocardiogram revealed no significant abnormalities. Imaging studies showed mild bilateral hemopneumothorax and a splenic laceration. Skeletal injuries included an aligned sternal body fracture and multiple bilateral rib fractures, without evidence of instability. Posterior bone fragments from the fractures of the left 5th to 7th ribs were in contact with the posterior wall of the descending thoracic aorta, with a minimum separation of 2 mm (Fig. 1), posing a risk of vascular injury. Given this context, the patient underwent successful surgical excision of the bone fragments by the thoracic surgery team.

